# TNFα expressed on the surface of microparticles modulates endothelial cell fate in rheumatoid arthritis

**DOI:** 10.1186/s13075-018-1768-8

**Published:** 2018-12-07

**Authors:** Cristiana Barbati, Marta Vomero, Tania Colasanti, Marco Diociaiuti, Fulvia Ceccarelli, Sara Ferrigno, Annacarla Finucci, Francesca Miranda, Lucia Novelli, Carlo Perricone, Francesca Romana Spinelli, Simona Truglia, Fabrizio Conti, Guido Valesini, Cristiano Alessandri

**Affiliations:** 1 Arthritis Center, Department of Internal Medicine and Medical Specialties, Sapienza University of Rome, Rome, Italy; 2 Technology and health Department, Istituto Superiore di Sanità, Rome, Italy

**Keywords:** Rheumatoid arthritis, Microparticles, Autophagy, Endothelial cells

## Abstract

**Background:**

Rheumatoid arthritis (RA) is associated with a high prevalence of atherosclerosis. Recently increased levels of microparticles (MPs) have been reported in patients with RA. MPs could represent a link between autoimmunity and endothelial dysfunction by expressing tumor necrosis factor alpha (TNFα), a key cytokine involved in the pathogenesis of RA, altering endothelial apoptosis and autophagy. The aim of this study was to investigate TNFα expression on MPs and its relationship with endothelial cell fate.

**Methods:**

MPs were purified from peripheral blood from 20 healthy controls (HC) and from 20 patients with RA, before (time (T)0) and after (T4) 4-month treatment with etanercept (ETA). Surface expression of TNFα was performed by flow cytometry analysis. EA*.*hy926 cells, an immortalized endothelial cell line, were treated with RA-MPs purified at T0 and at T4 and also, with RA-MPs in vitro treated with ETA. Apoptosis and autophagy were then evaluated.

**Results:**

RA-MPs purified at T0 expressed TNFα on their surface and this expression significantly decreased at T4. Moreover, at T0 RA-MPs, significantly increased both apoptosis and autophagy levels on endothelial cells, in a dose-dependent manner*.* RA-MPs did not significantly change these parameters after 4 months of in vivo treatment with ETA.

**Conclusions:**

Our data demonstrate that MPs isolated from patients with RA exert a pathological effect on endothelial cells by TNFα expressed on their surface. In vivo and in vitro treatment with ETA modulates this effect, suggesting anti-TNF therapy protects against endothelial damage in patients with RA.

**Electronic supplementary material:**

The online version of this article (10.1186/s13075-018-1768-8) contains supplementary material, which is available to authorized users.

## Background

Microparticles (MPs) are small membrane vesicles (0.1–1.0 μm) released by many cell types under physiological and pathological conditions. In the past these particles were considered as inert cell debris, but recently many studies have demonstrated they could be involved in intercellular communication. Generation and scattering of MPs occurs during different biological processes, including apoptosis and cellular activation [1–3]. Due to their formation, MPs have an array of surface markers derived from their parental cell that can be used to assess their origin. Thus, MPs can transfer biological messages from parental to target cells by direct interaction with the ligands expressed on the surface of target cells and activate cascade signaling; or they can transfer proteins, messenger RNA (mRNA), micro RNA (miRNA), and bioactive lipids by fusion or internalization with target cells [4].Thus, MPs are able to modulate various biological phenomena such as cell proliferation, angiogenesis, immune response, and coagulation [5, 6].

Increased levels of MPs have been reported in various pathological conditions including infections, malignancies, and autoimmune diseases, such as rheumatoid arthritis (RA) [7]. RA is an autoimmune systemic inflammatory disease characterized by chronic synovial inflammation, resulting in cartilage and bone damage with accelerated atherosclerosis and increased mortality [8]. Tumor necrosis factor alpha (TNFα) is the main cytokine involved in the pathogenesis of RA and many studies agree on the pro-atherogenic effect of TNFα in patients with RA. TNF-inhibitors are effective treatments for joint inflammation in RA; however, very little is known about their effect on atherosclerosis and endothelial dysfunction, which occur in this disease. Previous studies have shown that TNF-inhibitors can improve endothelial function and decrease cardiovascular events in responder patients, highlighting the pro-atherogenic effect of TNFα in RA [9, 10].

According to the literature, MPs could also have a role in endothelial dysfunction, contributing to atherosclerosis in patients with RA [11]. Moreover, an imbalance between apoptosis and autophagy mechanisms seems to be involved in endothelial dysfunction. Apoptosis is programmed cell death and many studies suggest the involvement of endothelial apoptosis in the atherosclerosis process [12]. Autophagy is a reparative process by which cytoplasmic components are sequestered in double-membrane vesicles and degraded on fusion with lysosomal compartments. It has been shown that basal autophagy is essential to proper vascular function [13].

Taking into account these considerations, the aim of this study was to analyze MP subsets in patients with RA and their contribution to endothelial dysfunction, with special focus on apoptosis and autophagy pathways, and to investigate if biological therapy could modulate these effects.

## Materials and methods

### Patients and controls

We enrolled 20 patients with RA fulfilling the 2010 American College of Rheumatology RA criteria [14], recruited from the Rheumatology Unit of Sapienza University of Rome at time zero (T0) and after 4 months (T4) of therapy with the TNF-inhibitor etanercept (ETA). The main clinical and laboratory variables assessed were the erythrocyte sedimentation rate (ESR), C-reactive protein (CRP), rheumatoid factor (RF) and anti-cyclic citrullinated peptide (anti-CCP) titer, tender and swollen joint count, patient’s assessment of pain, patient’s assessment of disease activity, physician’s global assessment of disease activity, and the health assessment questionnaire (HAQ). The European League Against Rheumatism (EULAR) response to therapy was also recorded before and after treatment. All patients had a history of failed treatment with at least one disease-modifying antirheumatic drug (DMARD). Patients were allowed to continue DMARDs, steroids, and non-steroidal anti-inflammatory drugs at a stable dose for at least 4 weeks before and during ETA treatment. Patients received ETA at a dose of 50 mg given by subcutaneous injection (sc) weekly. Methotrexate (MTX) was given at a dose of 10–20 mg weekly. In addition to MTX, hydroxychloroquine (200–400 mg daily) and steroids (maximum daily dose 10 mg of oral prednisone or equivalent) were also permitted.

Simultaneously, 20 healthy controls (HC) within a similar age range and gender as the patients were recruited. Also 10 patients with RA who had only been treated with traditional DMARDs were enrolled.

The protocol of study was approved by the Ethics Committee of Sapienza University of Rome (Protocol number 109/18), and informed written consent was obtained from all patients prior to enrollment.

### Isolation of MPs

A fasting blood sample was obtained from patients with RA, at T0 and T4, and from HC by venipuncture in 5-ml tubes containing citrate, which were centrifuged two times at 2500 g for 15 min at room temperature, to obtain platelet-poor plasma (PPP), rich in MPs. The resulting plasma was divided into five aliquots and stored at − 80 °C until analysis [15, 16].

### Energy-filtered transmission electron microscopy (EF-TEM)

For electron microscopy analysis MPs were centrifuged at 14000 rpm for 1 h at 4 °C and pellets were fixed. The samples were observed using a Zeiss EM902 transmission electron microscope, operating at 80 kV and equipped with an “in column” electron energy filter. The filter was set to collect only elastic electrons (ΔE 1/4 0), with the result of enhancing image contrast and resolution, thanks to the elimination of inelastic electrons in the image formation (reduction of the chromatic aberration). The sample was stained with 2% (*w*/*v*) phosphotungstic acid (PTA) in buffered aqueous solution at pH 7.3 (NaOH), previously filtered by polycarbonate 0.2-μm filters to eliminate impurities. Images were acquired with a digital charge-coupled device camera, model PROSCAN HSC2 (1 K × 1 K pixels), thermostated by a Peltier unit. The analysis was performed using a digital analyzer SIS 3.0. The overall resolution is in the range of 2 nm [16].

### Assessment of MP subsets and surface expression of TNFα by flow cytometry

MPs were analyzed using a FACS Calibur cytometer (Becton Dikinson, BD). Forward scatter (FSC) and side scatter (SSC) were adjusted to logarithmic gain. MP gating was accomplished using 1-μm beads (SIGMA) for setting upper limits in both FSC and SSC signals, and a lower limit was placed to exclude buffer noise.

Antibodies anti-CD41a–(Percp), anti-CD45–(APC) anti-CD31–(PE) were added to MP samples to identify specific MP subsets: platelet MPs (PMP (CD41a^+^ CD31^+^)), leukocyte MPs (LMP (CD45^+^)) and endothelial MPs (EMP (CD41a^−^ CD31^+^)) [17]. To evaluate the surface expression of TNFα, MPs were incubated with antibodies anti-TNFα–(fluorescein isothiocyanate (FITC)). MPs were also labeled using FITC-conjugated Annexin-V (AV). Fluorescent-conjugated isotype mAbs were used as controls. Incubation was performed at room temperature for 30 min in AV buffer and then MP suspensions were transferred into count tubes that were immediately processed by flow cytometry. MP number was calculated as described [18, 19]. All antibodies were purchased from BD Biosciences, San Josè, CA, USA.

### RA-MP incubation with ETA

In vitro treatment of MPs was performed with ETA. Briefly, 2*10^6^ MPs/ml RA-MPs purified at T0 were cultured in Dulbecco’s modified Eagle’s medium (DMEM) containing 10% fetal bovine serum (FBS), and treated with ETA at different concentrations (1, 3, 5, and 10 μg/ml) for 30 min, 2 h and 4 h. Surface expression of TNFα was evaluated at the end of each time period.

### In vitro culture of EA*.*hy926 cells

The in vitro effects of plasma-isolated MPs on the endothelium were evaluated using human umbilical vein cell line EA*.*hy926. Cells were cultured in DMEM containing 10% FBS, 50 IU/ml penicillin, 50 μg/ml streptomycin and 2 mM l-glutamine at 37 °C under an atmosphere of 5% CO2. To this end, MP suspensions purified from patients with RA at T0 and T4 and from HC were prepared. Briefly, MPs were centrifuged at 14000 rpm for 1 h at room temperature and resuspended in DMEM 10% FBS to obtain the desired concentration. Cells were cultured with MP suspensions at different concentrations (0.5, 2 and 8*10^6^ MPs/ml) for 6 h, 16 h, 24 h, 48 h, and 72 h. Cells were also treated with RA-MPs pre-incubated with ETA. Where indicated, cells were treated in the presence of lysosomal inhibitors E64d and pepstatin A (PepA) (both at 10 μg/ml; Sigma-Aldrich) for 2 h before the end of the culture. For inhibition of autophagy, cells were treated with 10 mM 3-methyladenine (3MA), which pharmacologically blocks this catabolic process (Sigma-Aldrich). Apoptosis and autophagy were evaluated at the end of experiments.

### Evaluation of EA*.*hy926 cell apoptosis

After in vitro treatment with MPs, EA*.*hy926 cell apoptosis was analyzed by flow cytometry using a FITC-conjugated Annexin V and propidium iodide apoptosis detection kit [20] according to the manufacturer’s instructions (MBL).

### Analysis of autophagy and TNF pathway by western blot

For analysis of autophagy EA*.*hy926 lysates (30 μg) were loaded on SDS-PAGE and expression levels of the autophagic markers LC3-II and P62 were analyzed by western blot [21]. Rabbit anti-human p65 antibody was used for NF-kB-p65 protein detection,. All antibodies were purchased from Cell Signaling Technology, Beverly, MA, USA.

### Statistical analysis

Normal distribution of variables was assessed using the Kolmogorov-Smirnov test. Statistical analysis was performed using the program GraphPad Prism Version 6 (GraphPad Software, San Diego, CA, USA). The Mann–Whitney unpaired test or Student’s *t* test was used to compare quantitative variables in different groups. Statistical correlation was examined using Spearman’s rank correlation coefficient. Values of *p* < 0.05 were considered statistically significant.

## Results

### Patients and controls

The demographic, serological and clinical characteristics of patients with RA and of HC are summarized in Table 1.

**Table 1:** Clinical, demographic, serological, and therapeutic characteristics of patients with RA and HC

	Patients with RA (*n* = 20)	HC (*n* = 20)
Demographic parameters		
Female/male	19/1	20/0
Age, years (median (IQR))	58.5 (50–67)	58 (50–65)
Disease duration, years (mean ± SD),	5.3 ± 5.6	
Disease activity		
DAS28 (mean ± SD)	4.1 ± 1.7	
TJ, *n* (mean ± SD)	6.16 ± 5.9	
SJ, *n* (mean ± SD)	2.79 ± 2.7	
CDAI (mean ± SD)	18.8 ± 10.7	
HAQ (mean ± SD)	1.15 ± 0.8	
Laboratory parameters	N (%)	
RF+	15 (75)	
ACPA+	15(75)	
ESR, mm/h (mean ± SD)	16.5 ± 10.3	
CRP, mg/dl (mean ± SD)	0.7 ± 0.73	
Therapy		
Concurrent csDMARDs (*n* (%))	20 (100)	
ETA (*n* (%))	20 (100)	

*RA* rheumatoid arthritis, *HC* healthy controls, *SD* standard deviation, *DAS28* Disease Activity Score in 28 joints, *TJ* tender joints, *SJ* swollen joints, *CDAI* Clinical Disease Activity Index, *HAQ* Health Assessment Questionnaire, *RF* rheumatoid factor, *ACPA* anti-citrullinated peptide antibodies, *ESR* erythrocyte sedimentation rate, *CRP* C-reactive protein, *csDMARDs* conventional synthetic disease-modifying antirheumatic drugs

### Electron microscopy

**Fig. 1:**
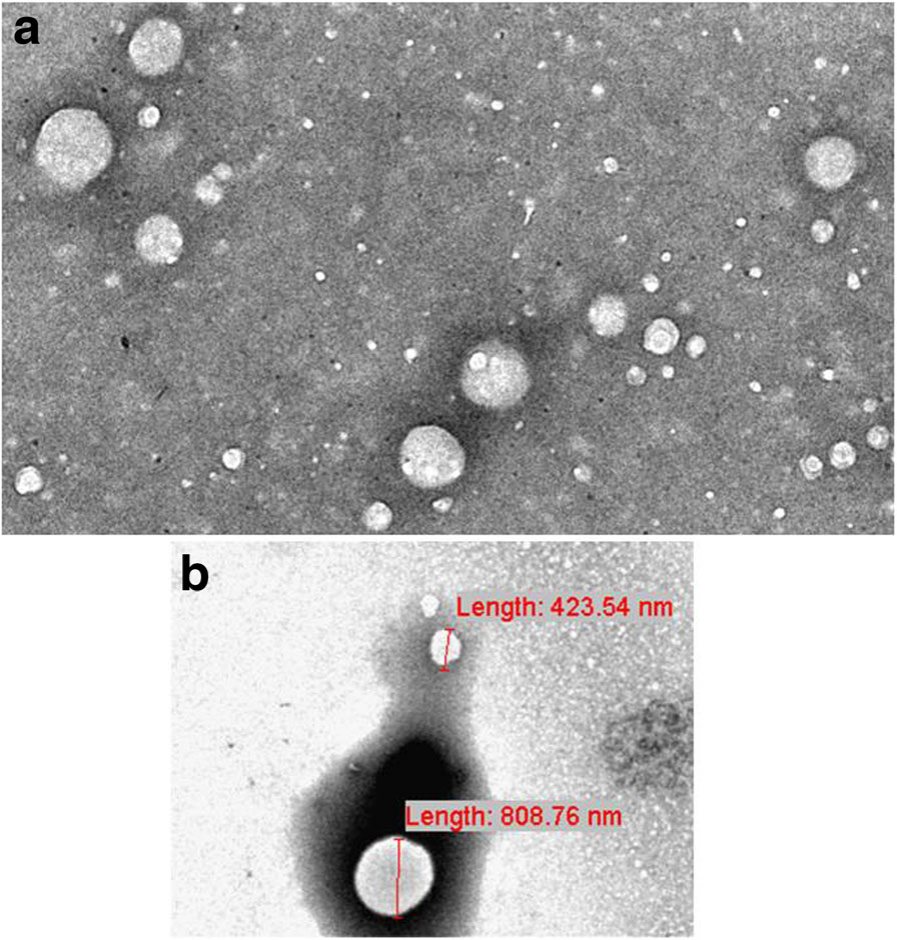
Transmission electron microscopy (TEM) of microparticles (MPs). Micrographs show the negative staining of a typical sample observed by the TEM. **a** White particles of different sizes ranging from a few nanometers to 1 mm were present. As expected, all particles appeared as white structures in the dark background, due to phosphotungstic acid (PTA) negative staining. They were round-shaped and sometimes superimposed. This observation suggests that the smallest nanoparticles and MPs coexist in our sample, because it is well-known that MP size ranges from 100 to 1000 nm. **b** Two MPs of about 500 nm in diameter are shown, together with their measured diameters. (Images from Miranda F. PhD Thesis)

Transmission electron microscopy of PPP confirms the purity of the samples used to conduct all our in vitro experiments. The images show vesicles that are heterogeneous in terms of shape and density, with the majority in the range of between 0.2 μm and 1 μm. Those sizes represent the typical size of MPs that differ from exosomes and apoptotic bodies in size, composition and mechanism of formation (Fig. 1).

### Evaluation of MP subsets and surface expression of TNFα

**Fig. 2:**
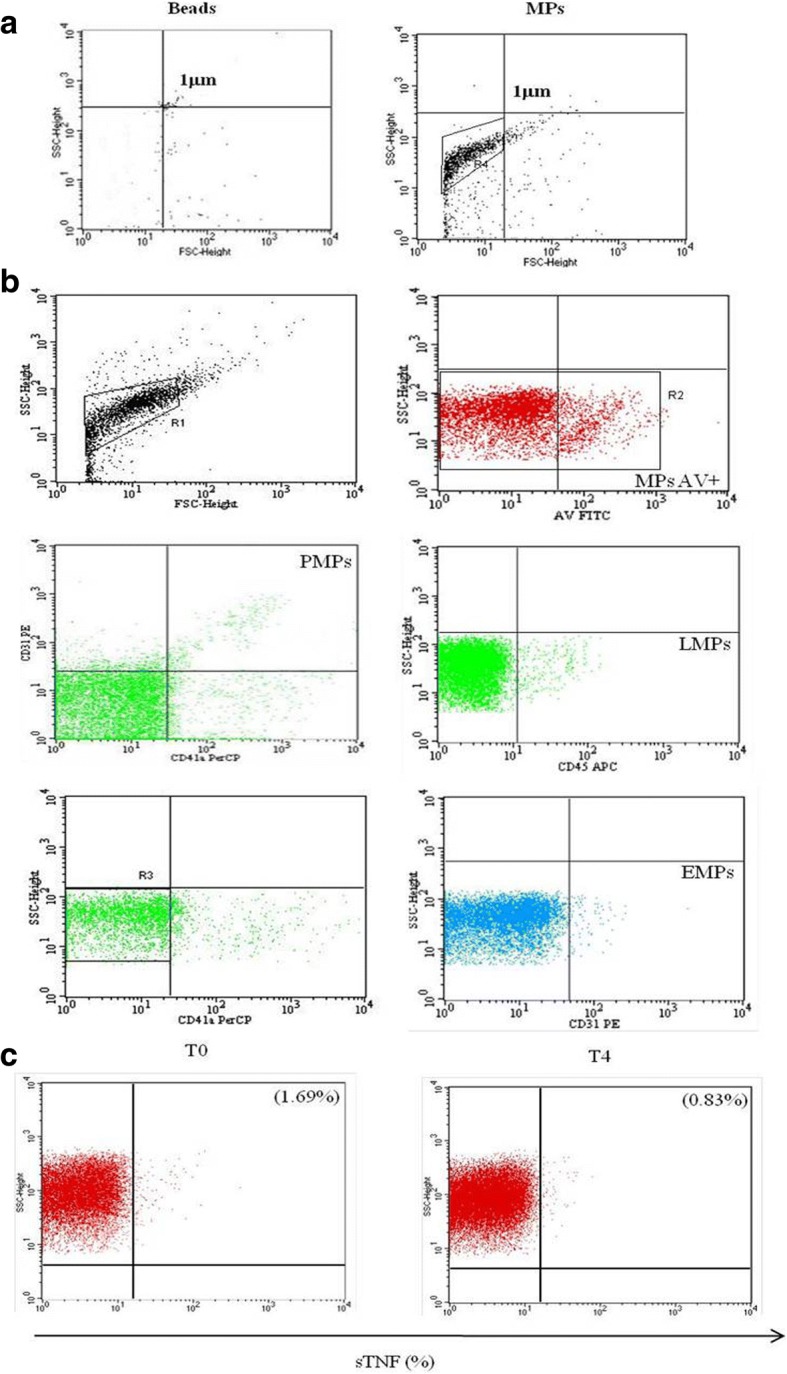
Gating strategy for flow cytometric analysis. **a** All microparticles (MPs) were gated as described in “Materials and methods”. **b** Flow cytometry analysis of specific binding of antibodies to MPs derived from platelets, leukocytes and endothelial cells and annexin-V (AV). **c** MPs were also labeled for the surface expression of TNFα. SSC, side scatter; FSC, forward scatter; FITC, fluorescein isothiocyanate; PMP, platelet MPs; LMP, leukocyte MPs; EMP, endothelial MPs; APC, allophycocyanin; PE, phycoerythrin; PerCP, peridin chlorophyll protein; T0, time zero (baseline); T4, 4 months after treatment

The number of MPs in serum from patients with RA and HC was quantified by flow cytometry analysis. The strategy used to identify MP morphology, MP subsets and TNFα expression is shown in Fig. 2a, b, and c.

**Fig. 3:**
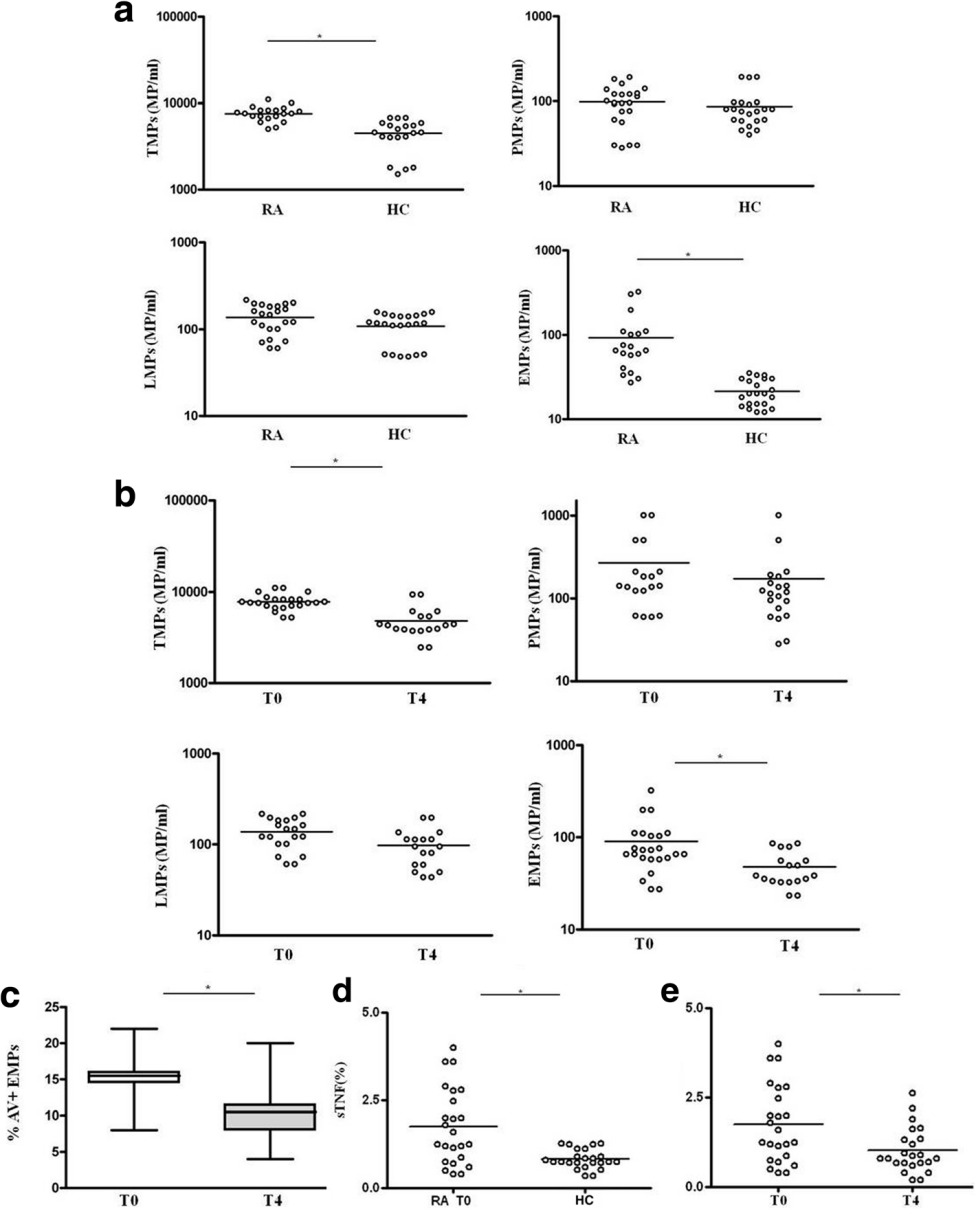
Plasma levels of microparticles (MPs) in patients with rheumatoid arthritis (RA) at time zero (baseline (T0)) and after 4 months of treatment (T4) and in healthy controls (HC) and surface expression of TNFα. **a** Relative distribution of total MPs (TMPs), platelet MPs (PMPs), leukocyte MPs (LMPs) and endothelial MPs (EMPs) in 20 patients with rheumatoid arthritis (RA) and 20 healthy controls (HC). **b**, **c** Relative distribution of TMPs, PMPs, LMPs, EMPs (**a**) and EMPs annexin-V (AV)+ (**c**) in patients with RA at time zero (baseline (T0) and 4 months after treatment (T4). **d** Relative distribution of the percentage of the expression of TNFα on the surface of MPs from patients with RA and HC. **e** Relative distribution of TNFα expression on MPs from patients with RA at T0 and T4. Each data point represents a single subject; horizontal lines show the median. Groups were compared using Student’s *t* test; *p < 0.05

Our study showed that at baseline the total number of MPs was significantly increased in patients with RA compared to HC (*p* < 0.0001) (Fig. 3a). Only the EMP subset was significantly increased in patients with RA compared to HC (*p* = 0.0049) (Fig. 3a). Interestingly, after 4 months of in vivo treatment with ETA, the total number of MPs and of the EMPs were both significantly decreased compared to baseline (*p* < 0.0001 and *p* = 0.03, respectively) (Fig. 3b). Moreover, after in vivo treatment with ETA, the percentage of EMPs that were AV-positive decreased, demonstrating downregulation of apoptosis of endothelial cells in vivo (*p* = 0.03) (Fig. 3c). There were no differences between seropositive and seronegative patients with RA with regard to the levels of MPs (data not shown). As shown in Fig. 3d the percentage of TNFα expressed on the surface of MPs (sTNFα) was significantly increased in patients with RA compared to HC (*p* = 0.0009). After 4 months of in vivo treatment with ETA the percentage of sTNFα was significantly decreased with respect to baseline (*p* = 0.0002) (Fig. 3e). Instead, there was no significant change in the percentage of sTNFα-MPs after therapy in patients treated only with traditional DMARDs (data not shown).This result corroborated our hypothesis on the capacity of ETA to bind TNFα carried on the surface of the MPs, as explained in the next paragraph. Moreover, as shown in Additional file 1, in relation to the expression of sTNF on MP subsets, we observed that EMPs expressed a greater percentage of TNFα than PMPs. LMPs expressed a high percentage of TNFα but this was not significantly different to the percentage in the other subsets.

Finally, to compare the percentage of TNFα expressed on the surface of the MPs and the serum TNFα, we also performed ELISA of serum TNF on the same patients in whom we evaluated the surface expression of TNFα on MPs, as shown in Additional file 1.

### Surface expression of TNFα on MPs after incubation with ETA

We demonstrated for the first time the expression of TNF on the surface of human MPs. On the basis of this result we decided to incubate MPs with ETA at different concentrations in order to demonstrate that the drug was able to bind and inhibit surface MP-TNFα expression. ETA, a fully soluble, human dimeric fusion protein, functions as a TNF inhibitor by competitively binding to TNF and preventing its activation of the inflammatory cascade. ETA is a soluble form of the p75 receptor that inhibits TNFα, by blocking its interaction with cell-surface TNF receptors and is different from adalimumab and infliximab, which are monoclonal antibodies to TNF. Moreover, its peculiarity with respect to the other TNFα inhibitors, is that besides recognizing soluble TNFα, it also recognizes the membrane-related portion.

**Fig. 4:**
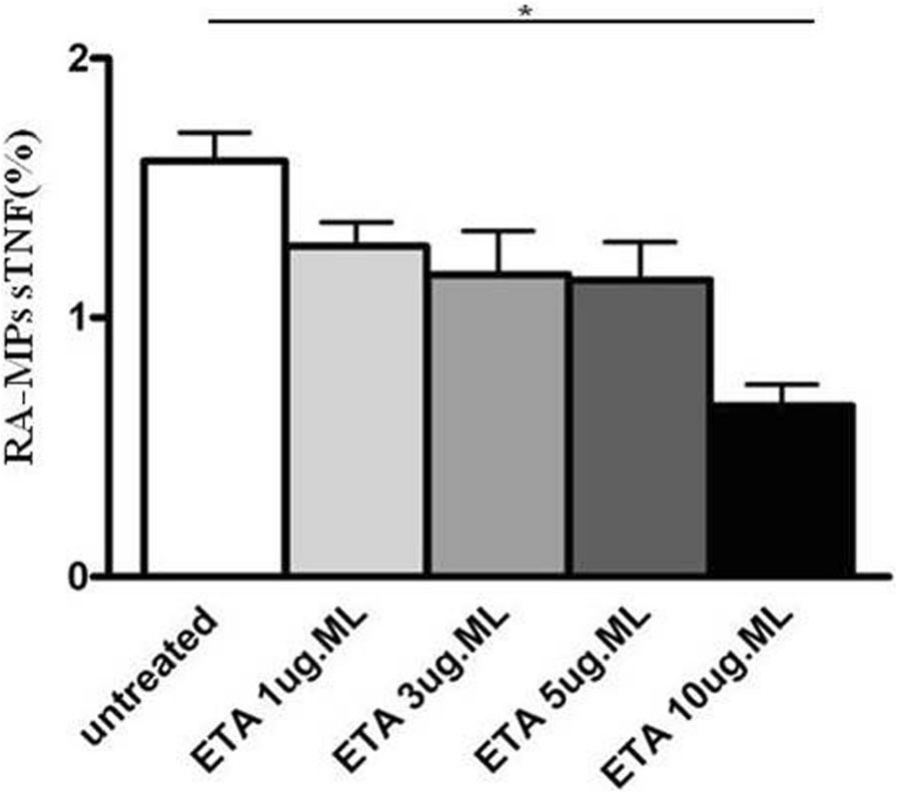
Surface expression of TNFα after incubation of rheumatoid arthritis (RA)-microparticles (MPs) with etanercept (ETA). Percentage of surface (s)TNFa expression on RA-MPs is shown after in vitro incubation with ETA at different concentrations for 2 h. Values are expressed as means ± SD; *p < 0.05

For this reason, we hypothesized that ETA, in addition to the soluble portion, recognizes and binds the portion transported on the membrane of the MPs, preventing the link with its receptor and then the inflammatory cascade. Results obtained confirmed our hypothesis, in fact, in vitro experiments showed that the percentage of sTNFα expressed on RA-MPs significantly decreased, in a dose-dependent manner, after incubation with ETA at each timepoint of the experiment. In particular, the major effect was at 10 μg/ml of ETA at (Fig. 4).

### The percentage of sTNFα correlates with clinical parameters of patients with RA

**Fig. 5:**
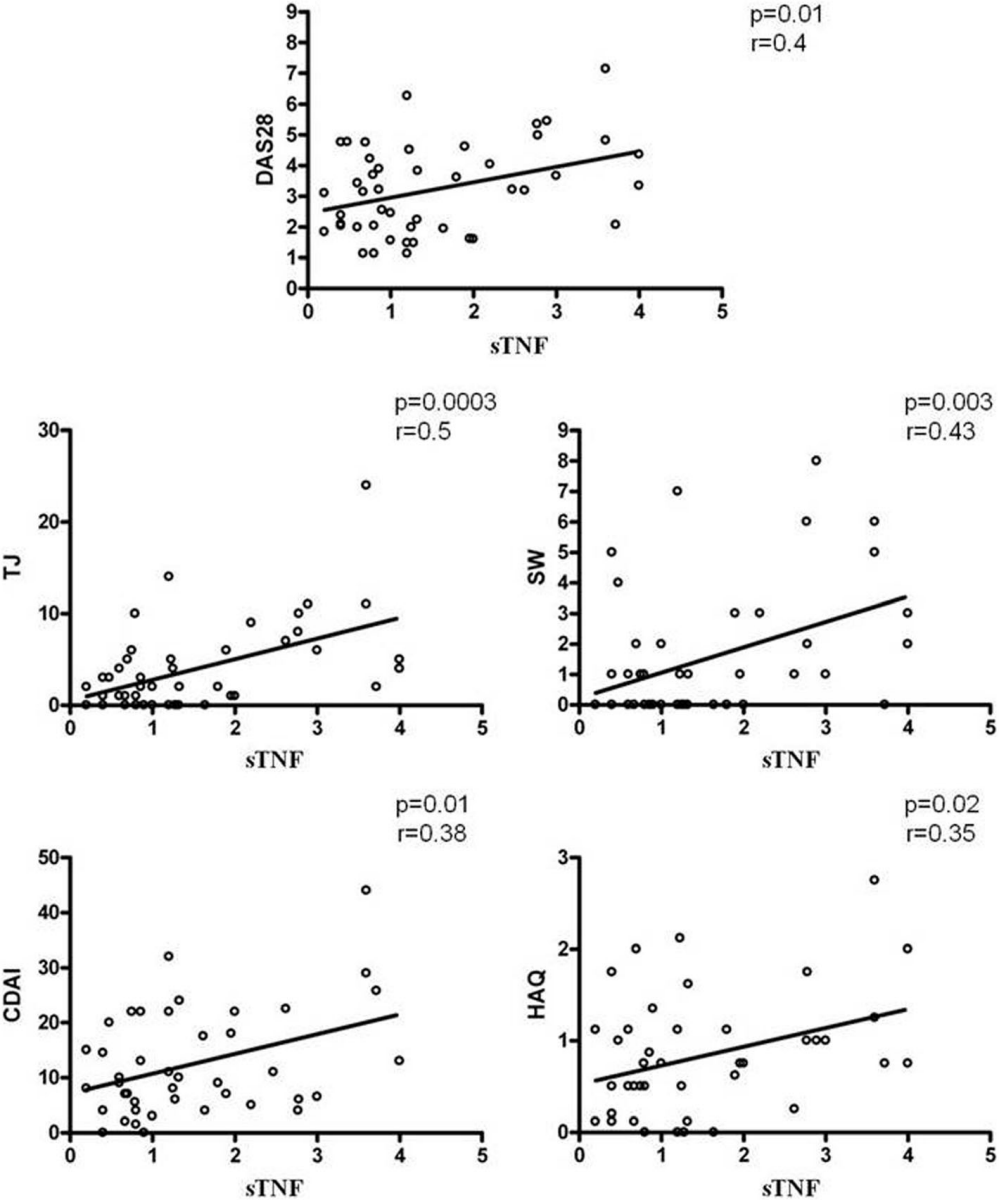
Correlation between the percentage of surface (s)TNFα expression on rheumatoid arthritis (RA)-microparticles (MPs) and clinical parameters of patients with RA. Correlation and linear regression analysis of sTNFα expression on MPs from patients with RA and Disease Activity Score in 28 joints (DAS 28), tender joints (TJ), swollen joints (SW), Clinical Disease Activity Index (CDAI) and Health Assessment Questionnaire (HAQ) (*r* and *p* values are shown on each diagram)

The purpose of our study was to test for correlation between MPs and endothelial damage. However, observing a decrease in the surface expression of MP-TNFα and a concomitant improvement in the parameters of disease activity in patients after therapy with ETA, we decided to test correlation between them. When we compared the sTNFα of RA-MPs and clinical parameters, such as the Disease Activity Score in 28 joints (DAS28), tender joints (TJ), swollen joints (SW), CDAI and HAQ, identified significant correlation between these parameters as shown in Fig. 5. These results lead us to suppose there is a link between sTNFα on MPs and the status of patients’ disease.

### sTNFα of RA-MPs mediates in vitro induction of apoptosis and autophagy in EA*.*hy926 cells

**Fig. 6:**
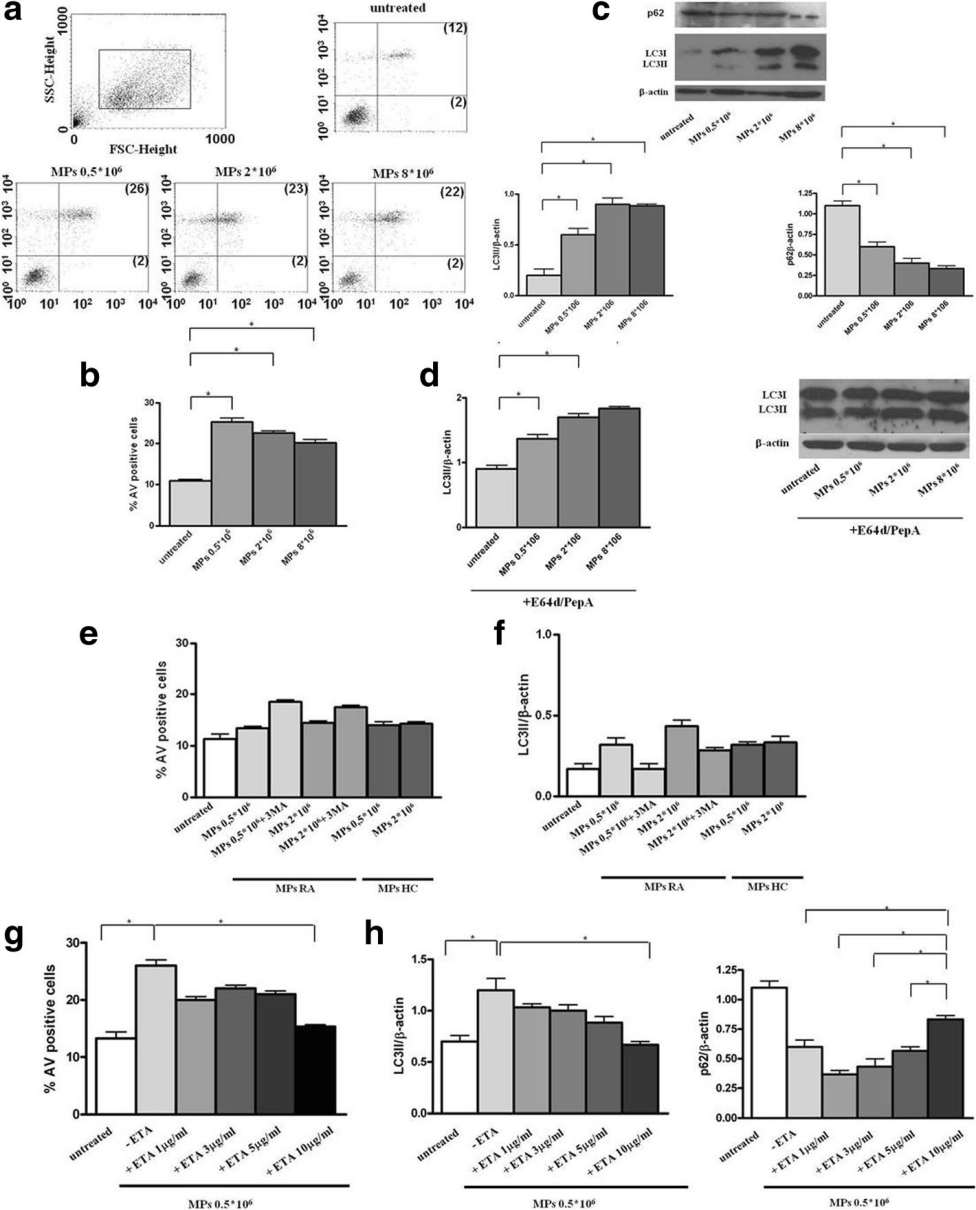
Induction of apoptosis and autophagy in EA.hy926 cells by microparticles (MPs) isolated from patients with rheumatoid arthritis (RA) after in vivo and in vitro treatment with etanercept (ETA) and by MPs isolated from healthy controls (HC). **a** Flow cytometry analysis of apoptosis in untreated MPs and MPs treated with EA.hy926 cells. Results obtained in a representative experiment are shown. Numbers in the upper and bottom right quadrants of each plot refer to annexin-V (AV)/propidium iodide (PI) double-positive cells and to AV single-positive cells, respectively. **b** Mean ± SD of the percentages of AV-positive cells obtained in three independent experiments; *p < 0.05. **c**, **d** Western blot analysis of LC3-II (left panel) and p62 (right panel) levels in EA.hy926 cells cultured with MPs purified from patients with RA at time zero (baseline (T0)), in the absence (**c**) or in the presence (**d**) of lysosomal inhibitors E64d and pepstatin A (PepA)**.** Blot shown is representative of three independent experiments. **e**, **f** Flow cytometry analysis of apoptosis (**e**) and western blot analysis (**f**) of LC3-II in EA.hy926 cells cultured with (i) MPs purified from patients with RA after 4 months of treatment with ETA, (ii) MPs purified from HC, and (iii) in the presence or absence of the autophagy inhibitor 3MA. **g**, **h** Flow cytometry analysis of apoptosis (**g**) and western blot analysis (**h**) of LC3-II (left panel) and p62 (right panel) levels in EA.hy926 cells cultured with MPs purified from patients with RA at T0 and with MPs from patients with RA at T0 in vitro-treated with ETA. **e**-**h** Mean ± SD of the percentages of AV-positive cells obtained in three independent experiments is reported and densitometry analysis of LC3-II and p62 levels relative to β-actin is shown. Values are expressed as means ± SD; *p < 0.05. FSC, forward scatter; SSC, side scatter

TNFα is the main cytokine involved in RA pathogenesis and has a pathogenic effect, with a direct effect on endothelial cells. Earlier studies have shown that TNFα inhibitors are able to improve endothelial function and decrease cardiovascular events in those patients who respond clinically [9, 10]. After demonstrating the expression of TNFα on the surface of MPs we decided to investigate whether MPs induce apoptosis and autophagy on endothelial cells and whether TNFα carried on their surface was responsible for this effect. Our in vitro results on EA*.*hy926 cells showed that at baseline (T0), serum RA-MPs significantly increased both apoptosis and autophagy levels compared to untreated cells, in a dose-dependent manner (*p =* 0.005 and *p =* 0.02*,* respectively*)* (Fig. 6a–d).

After 4 months of in vivo treatment with ETA, RA-MPs did not significantly change these parameters (*p* > 0.05 versus untreated cells for both parameters) (Fig. 6e, f). These effects were comparable to those obtained with the same number of MPs from HC (Fig. 6e, f). Interestingly, in the same experiment described above, treatment with the 3MA restored a situation comparable to that at T0, with an increase in endothelial apoptosis after autophagy block in EA*.*hy926 cells treated with MPs at T4, asserting the protective role of autophagy (Fig. 6e, f). The experiments reported above showed the results at 16 h, but a cellular response, comparable to that obtained at 16 h, had begun by 6 h when the cells were incubated with RA-MPs, (data not shown). However, at 24 h, 48 h, and 72 h the cellular response in terms of apoptosis and autophagy gave rise to confused and uninterpretable results, probably because, after some hours, other surface molecules started a cellular response, or the MPs fused themselves with the cells or were internalized, and so the molecular content of MPs may have altered the initial response (data not shown).

Moreover, in vitro studies showed that RA-MPs treated with ETA were no longer able to significantly modulate apoptosis and autophagy in EA*.*hy926 cells. In particular, when MPs were treated with ETA at 10 μg/ml, both endothelial apoptosis and autophagy reached levels comparable to those of untreated cells (*p* = 0.01 and *p* = 0.02, respectively versus cells treated with RA-MPs at T0), to confirm possible involvement of TNFα carried on MPs in the induction of apoptosis and autophagy (Fig. 6g, h).

**Fig. 7:**
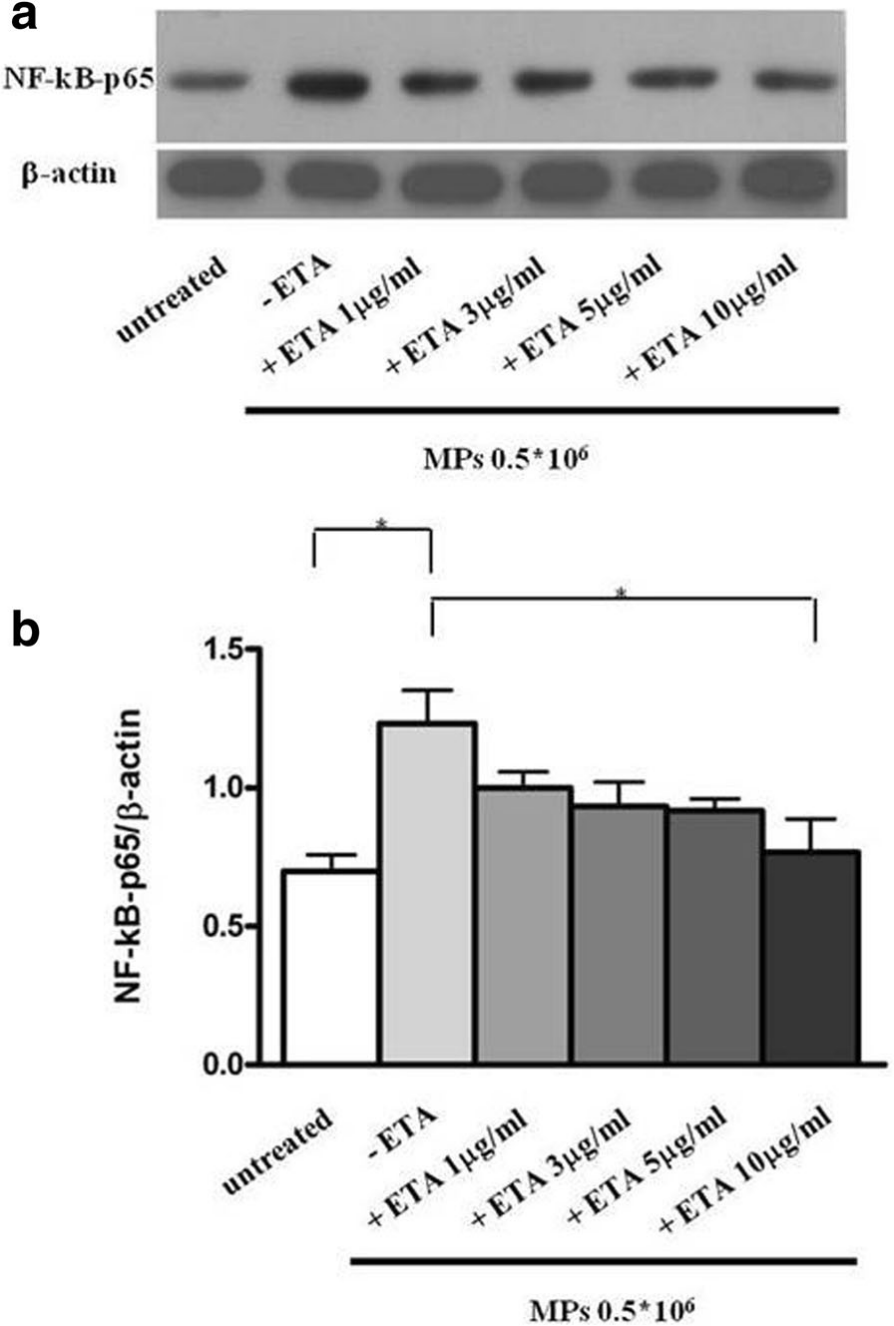
Activation of the TNFα pathway by microparticles (MPs)*.* **a** Western blot analysis of NF-kB levels in EA*.*hy926 cells cultured with MPs purified from patients with rheumatoid arthritis (RA) at time zero (baseline (T0) and with MPs from patients with RA at T0 in vitro-treated with etanercept (ETA). Blot shown is representative of three independent experiments. **b** Densitometry analysis of NF-kB relative to β-actin*.* Values are expressed as means ± SD; *p < 0.05

Finally, the result of western blot for NF-kB showed a significant decrease in p65 protein when endothelial cells were incubated with RA-MPs purified at T0 and treated with ETA at 10 μg/ml (*p* = 0.02 versus cells treated with RA-MPs at T0). This result confirms the hypothesis that MPs carry TNFα on their surface, which activates TNF-signaling pathways by interaction with its surface receptors (Fig. 7).

## Discussion

In our study, for the first time we demonstrated TNFα expression on the surface of MPs from patients with RA [22]. Higher circulating levels of TNFα are present in serum from patients with RA, and TNFα itself is able to directly damage endothelial function triggering NF-κB activation and accumulation of reactive oxygen species (ROS) [23].

Some evidence indicates a beneficial effect of TNF inhibitors on vascular wall physiology, increasing the possibility that TNF blockade may improve endothelial function, with consequent improvement of arterial stiffness and reduced progression of subclinical atherosclerosis [24, 25]. However, clinical studies conducted to investigate the effect of anti-TNF therapy on endothelial function in patients with RA have had contradictory results, generating controversy on this subject.

Based on our results and these considerations, we hypothesized that TNFα carried on the surface of RA-MPs could participate in the atherosclerotic process in patients with RA. Impaired endothelial cell function is a hallmark of atherosclerosis. The endothelial dysfunction is directly associated with the development of atherosclerosis, and it is present during all stages of the disease. Moreover, alterations in endothelial function precede morphological atherosclerotic changes [26]. Injury to the endothelium is believed to be a preliminary event in most vascular diseases [27]. Furthermore, those alterations could include an imbalance between apoptosis and autophagy pathways.

Endothelial apoptosis has been associated with multiple cardiovascular events that cause vascular wall damage and atherosclerotic plaque, and consequently it promotes vascular injury [28]. MPs have been shown to initiate cell death when applied to cultured primary rat brain microvascular endothelial cells (RBMVECs) and a variety of different mechanisms have been identified [29].

Previous studies showed that MPs derived from human umbilical vein endothelial cells [30] and platelets [31] contain caspase 3, and Schneider et al. [32] have suggested that circulating MPs induce apoptosis by the transfer of caspases into target cells. In relation to this, Abid Hussein et al. [30] suggest that MPs could be phagocytosed by cells, causing the release of caspase 3 within the cell itself and initiating apoptosis. In this study, treatment of MPs with a caspase 3 inhibitor significantly improved cell survival, indicating that the MPs impart pro-apoptotic caspase 3 signals.

Moreover, many studies showed that basal levels of autophagy are protective for the endothelium, continuously stimulated by mechanical and physical stress [33]. During injury, endothelial cell autophagy may occur to protect the cells from damage, while the failure of autophagy results in endothelial cell apoptosis, leading to the breakdown of the integrity of endothelium to induce the local lipid deposition into atheroma, plaque instability, and even acute coronary occlusion and death [34–37]. Nevertheless, the mechanisms that control the autophagy of endothelial cells are still limited. Moreover, autophagy has been shown to play an important role in endothelial cells to prevent development of atherosclerosis.

From the discovery of MPs up to now there has been no scientific evidence of the involvement of MPs in the regulation of autophagy pathway. Thus, in light of the numerous reported data, we can assume that MPs could be involved in alteration of apoptosis and autophagy, which together could contribute to the formation and progression of atherosclerotic plaque [38]. On the basis of this observation, after investigating surface expression of TNFα on MPs purified from patients with RA, we evaluated if MPs were able to modulate endothelial apoptosis and autophagy in vitro, and if TNFα carried on the surface of MPs could be involved in these cellular processes. In this regard Schock and coworkers identified three independent pro-apoptotic signals induced by MPs on RBMVECs cells: caspase 3 and activation of TNF-related apoptosis-inducing ligand (TRAIL) and TNF receptors. To support our last point on the induction of apoptosis by sTNF-MPs on EC, this study showed that treatment with a neutralizing antibody to TRAIL and TNF receptor blocker did improve cell survival. Genetic analysis confirmed that Tumor necrosis factor receptor 1 and 2 *(*TNFR1, TNFR2) and TRAIL receptor 4 are present in the cells. TRAIL may activate the extrinsic apoptotic pathway, but in some circumstances may activate pro-survival or proliferative pathways [29]. TNF is synthesized as a type-2 transmembrane protein and cleaved by the tumor necrosis factor-alpha-converting enzyme (TACE) ADAM17 to release soluble TNF, which may then activate TNF receptors. The authors showed that treating MPs with the TACE inhibitor (TAPI-0) prior to applying MPs to cultured cells provides a significant improvement in cell survival. This result indicates that TACE is present and active in MP membranes. Given the small size of MPs it is uncertain whether they are able to shed soluble TNF for an extended period of time.

According to these reported data, our results confirm that MPs from RA at T0 significantly increased apoptosis in endothelial cells. Interestingly, MPs purified from RA at T4, after in vivo treatment with ETA, and RA-MPs after in vitro treatment with ETA, were no longer able to significantly modify this parameter.

We obtained the same results for autophagy; indeed MPs obtained from RA at T0 significantly increased autophagy in endothelial cells and MPs purified from RA after in vivo treatment with ETA, and RA-MPs after in vitro treatment with ETA, were no longer able to significantly modify the autophagy pathway. The experiments with the 3MA, which is able to block autophagy and increase apoptosis of endothelial cells treated with RA-MPs, led us to suppose that MPs, if they induce cell death on one side, on the other side they induce cells to upregulate autophagy as a protective mechanism, at least at an early stage.

Furthermore, we demonstrated that RA-MPs induced an increase in NF-kB expression in endothelial cells, and that RA-MPs in vitro treated with ETA did not. These conclusive results support the concept, already proposed in other studies [39, 40], that the TNF transported on the surface of MPs interacts with TNF receptors on endothelial cells and activates the TNF-signaling cascade, including apoptosis and autophagy pathways.

Moreover, we observed significant correlation between DAS28 and TNFα expression on the surface of RA-MPs. This result supports other clinical observations that demonstrated the efficacy and safety of ETA in patients with active RA.

Finally, the interpretation of the ELISA results, i.e. that serum TNF does not change or increase after therapy, as demonstrated in other studies [41, 42], induced us to hypothesize the possibility of using surface expression of TNF on MPs as a possible therapy response marker. Obviously, to confirm this statement we need a far greater cohort of patients.

## Conclusions

Our results suggest that MPs isolated from patients with RA could exert their pathological effect on endothelial cells by TNFα expressed on their surface. In vivo treatment with ETA attenuates this effect, probably binding and blocking the TNF carried on the surface of MPs, as confirmed by our in vitro studies. To conclude, these results show a new pathway of endothelial damage mediated by MPs and confirm the protective effect of anti-TNF therapy against endothelial damage in patients with RA.

Certainly, we cannot assume that sTNF on MPs is the only architect of a cellular response; we hypothesize that it acts in synergy with multiple factors, some of which are already widely discussed in the literature, like serum TNF.

## Additional file

Additional file 1: Supplementary material and figures. (PDF 424 kb)

## Abbreviations

3MA: 3-Methyladenine
AV: Annexin-V
anti-CCP: Anti-cyclic citrullinated peptide
APC: Allophycocyanin
CDAI: Clinical Disease Activity Index
CRP: C-reactive protein
DAS28: Disease Activity Score in 28 joints
DMARD: Disease-modifying antirheumatic drug
DMEM: Dulbecco’s modified Eagle’s medium
EA*.*hy926: Human umbilical vein cell line
EF-TEM: Energy-filtered transmission electron microscopy
ELISA: Enzyme-linked immunosorbent assay
EMPs: Endothelial-derived microparticles
ESR: Erythrocyte sedimentation rate
ETA: Etanercept
FBS: Fetal bovine serum
FITC: Fluorescein isothiocyanate
FSC: Forward scatter
HAQ: Health Assessment Questionnaire
HC: Healthy controls
LMPs: Leukocyte-derived microparticles
mAbs: Monoclonal antibodies
MPs: Microparticles
PE: Phycoerythrin
PepA: Pepstatin A
PerCP: Peridin chlorophyll protein
PMPs: Platelet-derived microparticles
PTA: Phosphotungstic acid
RA: Rheumatoid arthritis
SJ: Swollen joints
SSC: Side scatter
STNFα: TNFα expressed on the surface of microparticles
T0: Time zero (before treatment)
T4: Four months after treatment
TJ: Tender joints
TNFα: Tumor necrosis factor alpha
