# Supplementary material for: TNFα expressed on the surface of microparticles modulates endothelial cell fate in rheumatoid arthritis
Source: Arthritis Res Ther. 2018 Dec 7;20:273. doi: 10.1186/s13075-018-1768-8 (PMC6286582; doi:10.1186/s13075-018-1768-8)
Supplement: Supplementary file 1 — Supplementary material and figures. (PDF 424 kb) [file 13075_2018_1768_MOESM1_ESM.pdf]

MATERIAL AND METHODS

Characterization of sTNF on MPs subsets

Antibodies anti-CD41a– (Percp), anti-CD45–(APC) anti-CD31–(PE) and anti-TNFα–(FITC) were added to MP samples to characterize the expression of sTNF on MPs subset by flow cytometry.

ELISA

An ELISA was performed to determine serum concentration of TNF in RA sera stored during patient's recruitment time. The commercial kit was performed following manufacturer's instruction (R&D System).

Correlation between TNFα and clinical parameters

The outcome of our analyses was to evaluate if sTNFα percentage (independent variable) was predictor of indexes of disease activity status: DAS28 or CDI or HAQ or SW or TJ, respectively. Analysis was performed by considering each variable at a time in a linear regression analysis model by using SPSS package.

RESULTS

Expression of sTNF on MPs subsets

After characterization of TNFα on the surface of MPs, we decided to perform the same analysis on MPs subset. So, our results showed that EMPs expressed a percentage of sTNFα that was significantly greater than those of PMPs (p=0.0115). LMPs expressed a high percentage of TNFα but not significantly different respect to the others subset (Fig. 1). So, the major source of sTNF-MPs seems to be, as expected, the endothelial portion.

Determination of serum TNF and correlation with sTNF-MPs

Results of ELISA test showed that at T0 and T4 RA patients showed serum TNF levels which didn't significantly correlate with the percentage of TNFα expressed on MPS (Fig. 2a,b). This result could be due to the fact that we use different methods and different units of measurement.

At T4, only 5 of the 20 patients tested showed a small decrease of serum TNFα respect to T0, while in the remaining cases it significantly increased ($p=0.006$)(Fig. 2c).

Instead, the TNF bound on the surface of the MPs significantly decreased after therapy with ETA ($p<0.0001$) (Fig. 2d). In light of these results we could hypothesize that the superficial expression of TNF on microparticles, unlike soluble TNF, could be used as a therapy response marker. Obviously to confirm this we need a far greater cohort of patients.

TNFα as predictor of indexes of disease activity status: DAS28 or CDI or HAQ or SW or TJ.

Although the cohort of RA patients was small, to confirm the results about correlation between sTNFα-MPs and clinical parameters described in the text, we re-analyzed our data reinforcing previous results, as shown in the supplementary Table 1.In addition, any single association was tested by bootstrap correction (P_{adj}-value) based on 1000 bootstrap samples, with the aim to adjust raw p-values and obtain more robust estimates of standard errors and confidence intervals of the TNFα values included in each model.

Supplementary Table 1

TNFα as predictor of indexes of disease activity status: DAS28 or CDI or HAQ or SW or TJ.

	R^2	R^2 Variation	F-test	*P*-value F-test	*β*	95% CI	*P*-value	P_{adj}-*value*
**TNF*α* *vs* DAS*28***	**0.14**	**0.14**	**7.149**	**0.010**	**0.37**	**0.12-0.87**	**0.010**	**0.008**
**TNF*α* *vs* CDI**	**0.29**	**0.29**	**16.837**	**<0.001**	**0.54**	**2.33-6.86**	**<0.001**	**0.002**
**TNF*α* *vs* HAQ**	**0.12**	**0.12**	**5.813**	**0.020**	**0.35**	**0.03-0.37**	**0.020**	**0.029**
**TNF*α* *vs* SW**	**0.18**	**0.18**	**9.568**	**0.003**	**0.42**	**0.29-1.37**	**0.003**	**0.013**
**TNF*α* *vs* TJ**	**0.27**	**0.27**	**15.926**	**<0.001**	**0.52**	**1.11-3.39**	**<0.001**	**0.005**

R^2-value was used to evaluate the percentage of variation of DAS28 or CDI, or HAQ, or SW, or TJ explained by TNFα.

β-coefficients values were used to evaluate the degree of change in DAS28 or CDI, or HAQ, or SW, or TJ for every 1-unit of change of TNFα.

F-test and P-value F-test were used to evaluate the significant amount of variance of R^2-variation of DAS28 or CDI, or HAQ, or SW, or TJ explained by TNFα variation.

P_{adj}-value was based on 1000 bootstrap samples.

Additional figure 1

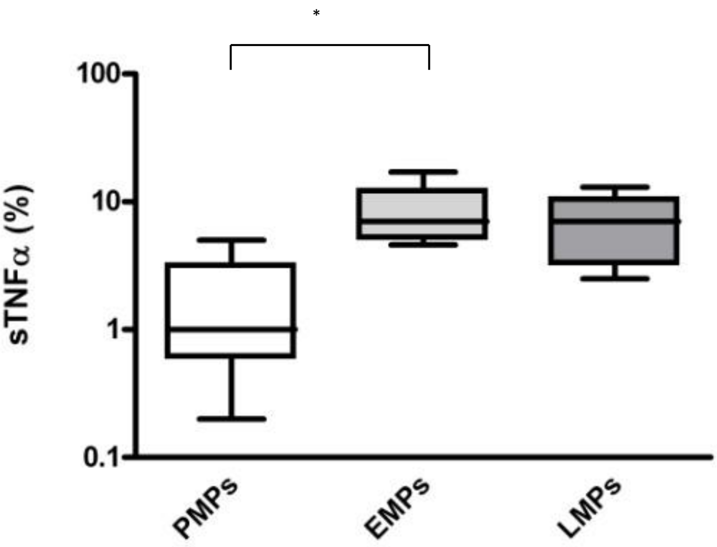

FIG.1. Expression of sTNF on MPs subsets.

Relative distribution of the percentage of the expression of TNF on the surface of RA-MPs subsets. Comparisons between groups were performed with Student’s t-test. **p* < 0.05.

Additional figure 2

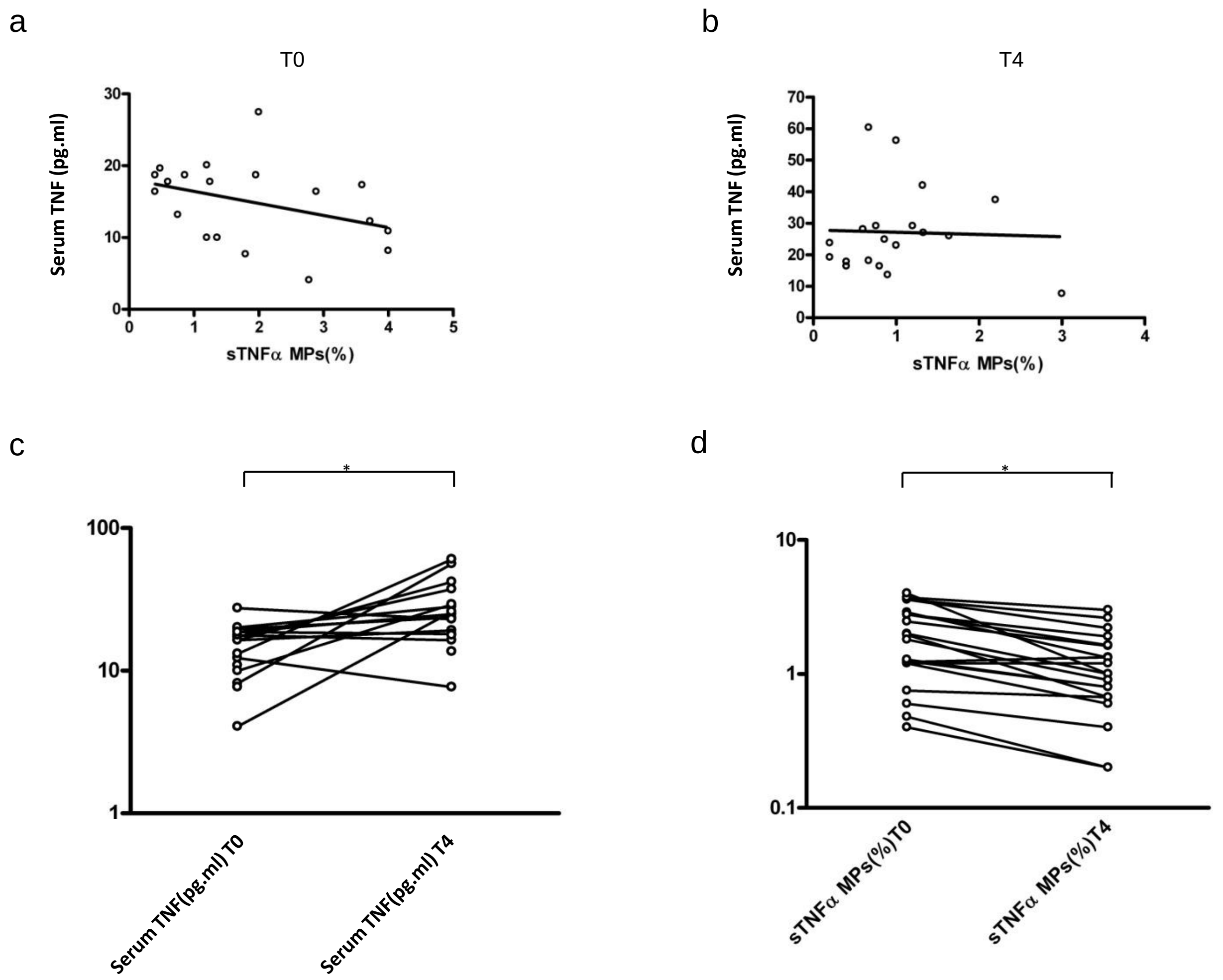

FIG.2: Determination of serum TNF and correlation with sTNF-MPs.
Correlation and linear regression analysis of serum TNF and sTNF expressed on MPs from RA patients at T0(**a**) and T4(**b**).
Graph at the left bottom shows the variation of serum TNF concentration in RA patients at T0 and T4(**c**). Graph at right bottom shows the change of percentage in TNF expression on the surface of the MPs in RA patients at T0 and at T4 (**d**).
Comparisons between groups were performed with Student's t-test. *$^*p < 0.05$.*
